# Supplementary material for: Time-series transcriptome analysis identified differentially expressed genes in broiler chicken infected with mixed Eimeria species
Source: Front Genet. 2022 Aug 8;13:886781. doi: 10.3389/fgene.2022.886781 (PMC9393255; doi:10.3389/fgene.2022.886781)
Supplement: Supplementary file 2 [file DataSheet1.ZIP › 7dpi_KEGG.Gsea.1625115961950/KEGG_HEMATOPOIETIC_CELL_LINEAGE.html]

Details for gene set KEGG\_HEMATOPOIETIC\_CELL\_LINEAGE[GSEA]

|  || Dataset | TMM\_7dpi\_gct\_format\_7dpi\_gct\_format.Class\_4dpi.cls #PC\_versus\_NC.Class\_4dpi.cls #PC\_versus\_NC\_repos |
| Phenotype | Class\_4dpi.cls#PC\_versus\_NC\_repos |
| Upregulated in class | 0 |
| GeneSet | KEGG\_HEMATOPOIETIC\_CELL\_LINEAGE |
| Enrichment Score (ES) | -0.48740956 |
| Normalized Enrichment Score (NES) | -1.5927069 |
| Nominal p-value | 0.004525911 |
| FDR q-value | 0.08305054 |
| FWER p-Value | 0.717 |
Table: GSEA Results Summary

  

Fig 1: Enrichment plot: KEGG\_HEMATOPOIETIC\_CELL\_LINEAGE      
 Profile of the Running ES Score & Positions of GeneSet Members on the Rank Ordered List

  

| SYMBOL | TITLE | RANK IN GENE LIST | RANK METRIC SCORE | RUNNING ES | CORE ENRICHMENT || 1 | CD55 | na | 1132 | 0.474 | -0.0750 | No |
| 2 | IL11RA | na | 1136 | 0.473 | -0.0556 | No |
| 3 | IL1B | na | 1237 | 0.445 | -0.0455 | No |
| 4 | CD1C | na | 1822 | 0.302 | -0.0817 | No |
| 5 | ITGA3 | na | 2012 | 0.270 | -0.0864 | No |
| 6 | CD4 | na | 2019 | 0.268 | -0.0757 | No |
| 7 | FCER2 | na | 2456 | 0.197 | -0.1040 | No |
| 8 | CD5 | na | 2505 | 0.191 | -0.1001 | No |
| 9 | TFRC | na | 2641 | 0.171 | -0.1043 | No |
| 10 | IL9R | na | 2743 | 0.158 | -0.1061 | No |
| 11 | ITGA2B | na | 2940 | 0.134 | -0.1170 | No |
| 12 | IL6R | na | 3564 | 0.056 | -0.1667 | No |
| 13 | CD14 | na | 3919 | 0.014 | -0.1957 | No |
| 14 | CD44 | na | 5024 | -0.106 | -0.2836 | No |
| 15 | CR1 | na | 5036 | -0.107 | -0.2801 | No |
| 16 | CD34 | na | 5318 | -0.132 | -0.2981 | No |
| 17 | CSF1 | na | 5321 | -0.133 | -0.2927 | No |
| 18 | ITGA4 | na | 5591 | -0.158 | -0.3087 | No |
| 19 | GP5 | na | 5607 | -0.160 | -0.3033 | No |
| 20 | ITGB3 | na | 5666 | -0.166 | -0.3013 | No |
| 21 | CD38 | na | 6007 | -0.202 | -0.3213 | No |
| 22 | CD1B | na | 6250 | -0.229 | -0.3320 | No |
| 23 | IL4R | na | 6643 | -0.269 | -0.3536 | No |
| 24 | CD9 | na | 6748 | -0.280 | -0.3507 | No |
| 25 | MME | na | 6754 | -0.280 | -0.3395 | No |
| 26 | IL1R1 | na | 8525 | -0.487 | -0.4672 | Yes |
| 27 | GP1BB | na | 8618 | -0.503 | -0.4540 | Yes |
| 28 | IL7 | na | 8686 | -0.511 | -0.4384 | Yes |
| 29 | CSF1R | na | 8919 | -0.538 | -0.4355 | Yes |
| 30 | CSF2RA | na | 8987 | -0.547 | -0.4184 | Yes |
| 31 | CSF3R | na | 9044 | -0.555 | -0.4000 | Yes |
| 32 | CD8A | na | 9773 | -0.669 | -0.4331 | Yes |
| 33 | ITGA1 | na | 9831 | -0.679 | -0.4097 | Yes |
| 34 | ANPEP | na | 10055 | -0.720 | -0.3984 | Yes |
| 35 | CD3D | na | 10250 | -0.758 | -0.3832 | Yes |
| 36 | ITGA6 | na | 10612 | -0.836 | -0.3787 | Yes |
| 37 | CD7 | na | 10660 | -0.845 | -0.3475 | Yes |
| 38 | IL2RA | na | 10774 | -0.872 | -0.3208 | Yes |
| 39 | CD36 | na | 10776 | -0.872 | -0.2847 | Yes |
| 40 | IL1R2 | na | 10937 | -0.917 | -0.2600 | Yes |
| 41 | KITLG | na | 10999 | -0.936 | -0.2263 | Yes |
| 42 | CD3E | na | 11250 | -1.017 | -0.2049 | Yes |
| 43 | ITGA2 | na | 11325 | -1.048 | -0.1677 | Yes |
| 44 | IL7R | na | 11582 | -1.161 | -0.1409 | Yes |
| 45 | CD2 | na | 11718 | -1.259 | -0.0999 | Yes |
| 46 | KIT | na | 11813 | -1.348 | -0.0519 | Yes |
| 47 | FLT3 | na | 11939 | -1.644 | 0.0059 | Yes |
Table: GSEA details [plain text format]

  

Fig 2: KEGG\_HEMATOPOIETIC\_CELL\_LINEAGE      
 Blue-Pink O' Gram in the Space of the Analyzed GeneSet

  

Fig 3: KEGG\_HEMATOPOIETIC\_CELL\_LINEAGE: Random ES distribution      
 Gene set null distribution of ES for **KEGG\_HEMATOPOIETIC\_CELL\_LINEAGE**
